# Directed acyclic graphs in perioperative observational research–A systematic review and critique against best practice recommendations

**DOI:** 10.1371/journal.pone.0281259

**Published:** 2023-02-09

**Authors:** Matthew Lamont Watson, Sebastian H. M. Hickman, Kaya Marlen Dreesbeimdiek, Katharina Kohler, Daniel J. Stubbs

**Affiliations:** 1 Clinical School of Medicine, University of Cambridge School of Clinical Medicine, Addenbrooke’s Hospital, Cambridge, United Kingdom; 2 Yusuf Hamied Department of Chemistry, University of Cambridge, Cambridge, United Kingdom; 3 The Alan Turing Institute, London, United Kingdom; 4 Department of Engineering, Health Systems Design Group, University of Cambridge, Cambridge, United Kingdom; 5 University Division of Anaesthesia, University of Cambridge, Cambridge, Addenbrooke’s Hospital, Cambridge, United Kingdom; 6 Wellcome Trust Research Fellow, University Division of Anaesthesia, Addenbrooke’s Hospital, Cambridge, United Kingdom; University Medical Center Göttingen: Universitatsmedizin Gottingen, GERMANY

## Abstract

The Directed Acyclic Graph (DAG) is a graph representing causal pathways for informing the conduct of an observational study. The use of DAGs allows transparent communication of a causal model between researchers and can prevent over-adjustment biases when conducting causal inference, permitting greater confidence and transparency in reported causal estimates. In the era of ‘big data’ and increasing number of observational studies, the role of the DAG is becoming more important. Recent best-practice guidance for constructing a DAG with reference to the literature has been published in the ‘Evidence synthesis for constructing DAGs’ (ESC-DAG) protocol. We aimed to assess adherence to these principles for DAGs constructed within perioperative literature. Following registration on the International Prospective Register of Systematic Reviews (PROSPERO) and with adherence to the Preferred Reporting Items for Systematic Reviews and Meta-Analyses (PRISMA) reporting framework for systematic reviews, we searched the Excerpta Medica dataBASE (Embase), the Medical Literature Analysis and Retrieval System Online (MEDLINE) and Cochrane databases for perioperative observational research incorporating a DAG. Nineteen studies were included in the final synthesis. No studies demonstrated any evidence of following the mapping stage of the protocol. Fifteen (79%) fulfilled over half of the translation and integration one stages of the protocol. Adherence with one stage did not guarantee fulfilment of the other. Two studies (11%) undertook the integration two stage. Unmeasured variables were handled inconsistently between studies. Only three (16%) studies included unmeasured variables within their DAG and acknowledged their implication within the main text. Overall, DAGs that were constructed for use in perioperative observational literature did not consistently adhere to best practice, potentially limiting the benefits of subsequent causal inference. Further work should focus on exploring reasons for this deviation and increasing methodological transparency around DAG construction.

## Introduction

The ability to ask causal questions of observational data is one of the core tasks of data analysis, alongside description and prediction [1]. Historically, healthcare researchers have refrained from explicitly seeking such findings [2], with an emphasis on the randomised controlled trial (RCT) as the only methodological framework capable of supporting such claims [1]. In many ways this was an understandable response to the potential for observational data analysis to suffer from various biases, including confounding and selection bias [3].

Since the late 1990s, frameworks for conducting robust, transparent causal research using observational data has emerged and become increasingly formalised across fields [4]. One of these frameworks represents variables of interest in a graphical format, underpinned by a robust mathematical framework [5]. These diagrams are termed directed acyclic graphs (DAGs) and consist of nodes (which represent the variables of interest) connected by directed edges (arrows). DAGs can be useful in a plethora of settings within healthcare research. This includes their use within interventional studies (such as RCTs), and observational research. To be valid for causal inference, DAGs must denote all causal relationships between nodes, specifically the root causes of any variable pair, even if these are unmeasured in available data [4]. Such unmeasured variables are termed ‘latent variables’. The use of DAGs allows transparent communication of a putative causal model between researchers and can prevent over-adjustment biases when conducting multivariable modelling. This could permit a greater degree of accuracy and confidence in calculated causal estimates.

Such a framework has clear benefits for perioperative and surgical researchers as healthcare data is continuing to grow [6], and perioperative care specifically generates a wealth of data pertaining to physiological, pharmacological, and broader perioperative events. A robust framework to seek causal inferences from this data is of huge benefit when studying relationships not amenable to an RCT [7]. An introduction to DAGs, their relevance to perioperative and surgical care, and the potential biases they can be used to identify has already been published [8, 9]. However, to our knowledge no study has yet sought to systematically catalogue the methods used to generate DAGs used in perioperative research and what problems they have been used to address.

This is, however, vital, as DAGs are increasingly used in other areas of health research [10]. The need for transparency and robustness in their generation is of increasing importance. A best practice framework for constructing DAGs has been recently published. The ‘Evidence synthesis for constructing DAGs’ (ESC-DAG) [11] protocol provides a recommended methodological approach to DAG construction. It consists of four main stages to build robust DAGs, drawing on previously published studies and containing clear instructions on how to synthesise this knowledge into a unifying causal model of the relationship under study. The ESC-DAG protocol contains four distinct stages. These stages include mapping (where relevant variables are identified from other literature studies), translation (where the relationships between variables are identified), integration 1 (where a single DAG is constructed with only the relevant relationships included) and integration 2 (where similar variables within the DAG are grouped together).

The primary aim of this systematic review is to provide the first comprehensive assessment of how DAGs are used and constructed within the perioperative literature, cataloguing the questions they are being used to address, their construction, and thus transparency. This was done by assessing DAG construction against the ESC-DAG protocol.

Our study also had two major secondary aims. Firstly, we examined how included studies handled unmeasured variables. This is vital as a DAG’s validity relies on its accurate inclusion of such latent variables [10]. Additionally, variable relationships within DAGs can also be modelled over time. This is becoming of increasing relevance given that electronic records can permit the repeated measurements of variables (such as blood results or physiological observations) through time. Therefore, our other secondary aim involved assessing how authors were incorporating time as a variable into their DAGs.

## Methods

This systematic review follows the Preferred Reporting Items for Systematic Reviews and Meta-Analyses (PRISMA) guidelines [12]. The 2020 PRISMA checklist can be found in the S1 Checklist. The aims and methods of our systematic review were registered prospectively on PROSPERO (CRD42021279183). No ethical approval was required for this study.

Published observational studies in the surgical and perioperative literature that had constructed a DAG were included in the study. This included observational studies that discussed any aspect of surgery or perioperative medicine.

**Fig 1:**
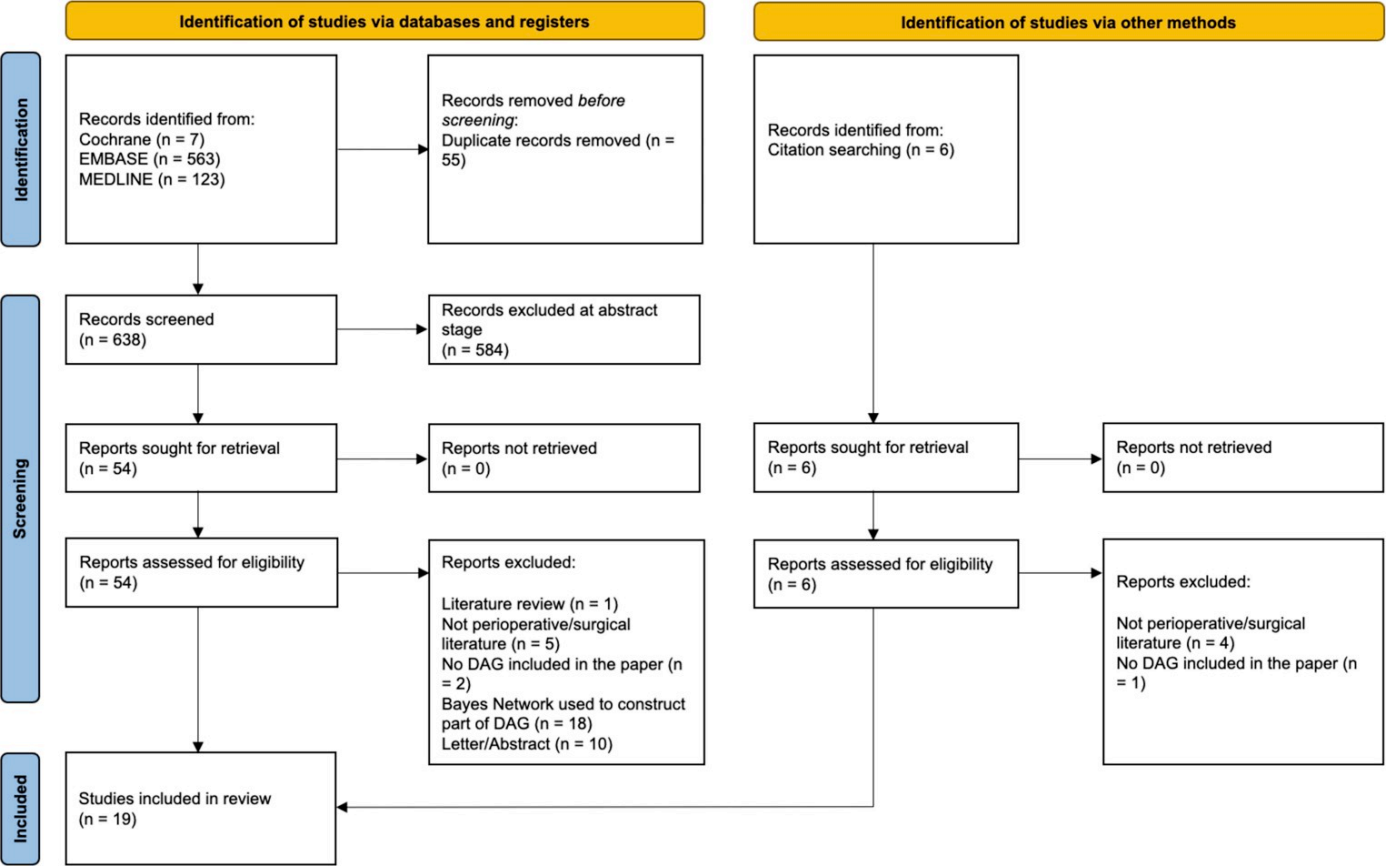
PRISMA flowchart of studies included in the final synthesis of the systematic review.

We searched Embase, MEDLINE and Cochrane databases. The search protocol for each database is available as S1 Appendix but broadly consisted of keywords relating to ‘Surgery’, ‘Anaesthesia’, ‘Perioperative’, and ‘Directed Acyclic Graph’. Following an initial search on the 7th of September 2021, we updated our search on 30th June 2022. The PRISMA flow chart is shown in Fig 1. All abstracts were screened independently by DS and MW. Abstracts were eligible for further full text screening if the title, abstract, or related keywords on the citation, suggested the study was a primary observational research study containing a DAG. This involved searching the abstract and titles for words such as ‘DAG’, ‘graph’, ‘acyclic’ and other language pertaining to causal inference. If the author was unsure, they were asked to include the abstract so the full text and supplementary material could be screened. If there were any disagreements, a third author (KK) was asked to adjudicate.

**Fig 2:**
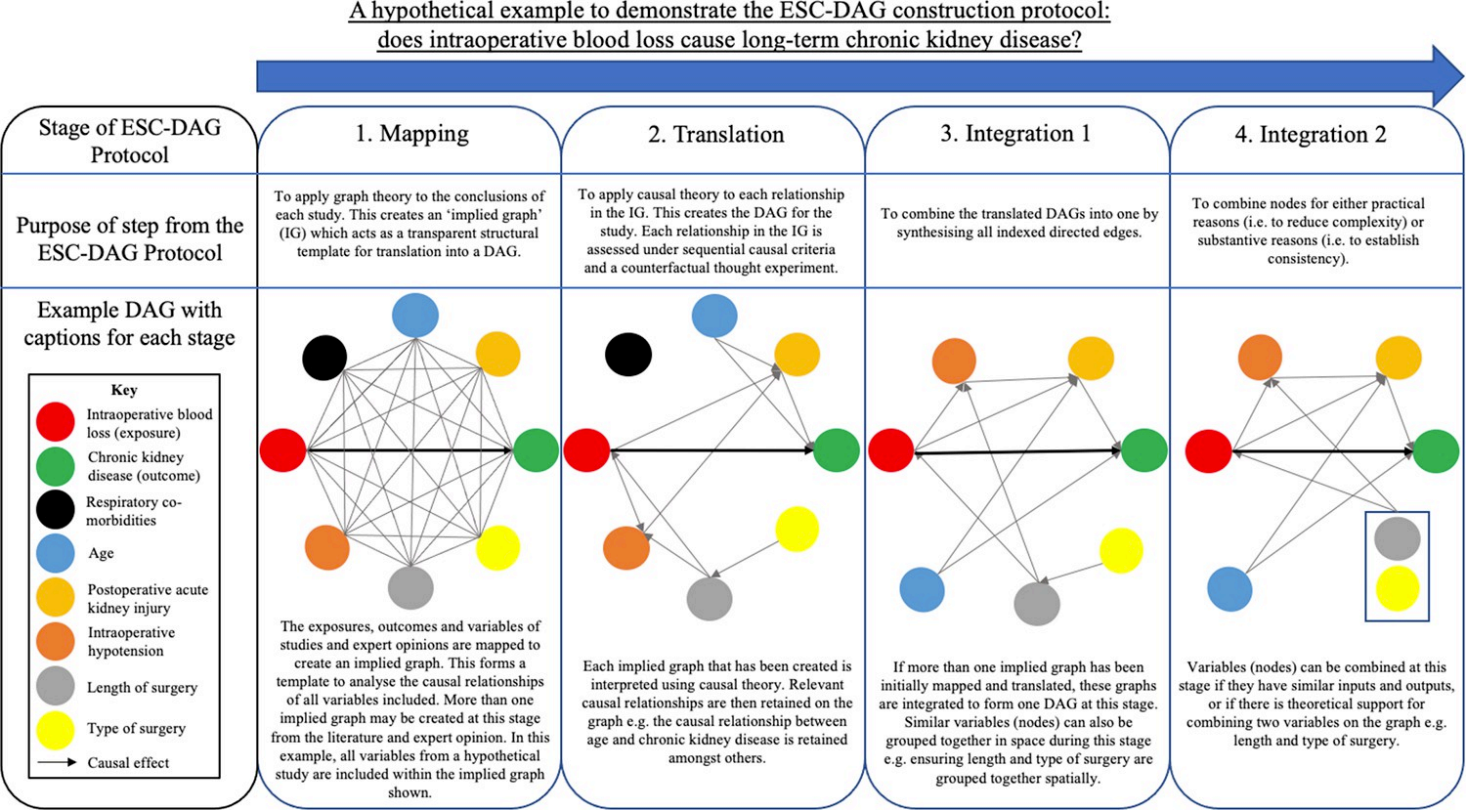
An outline of the stages of the ESC-DAG protocol [11], including an example construction of a perioperative DAG.

**Fig 3:**
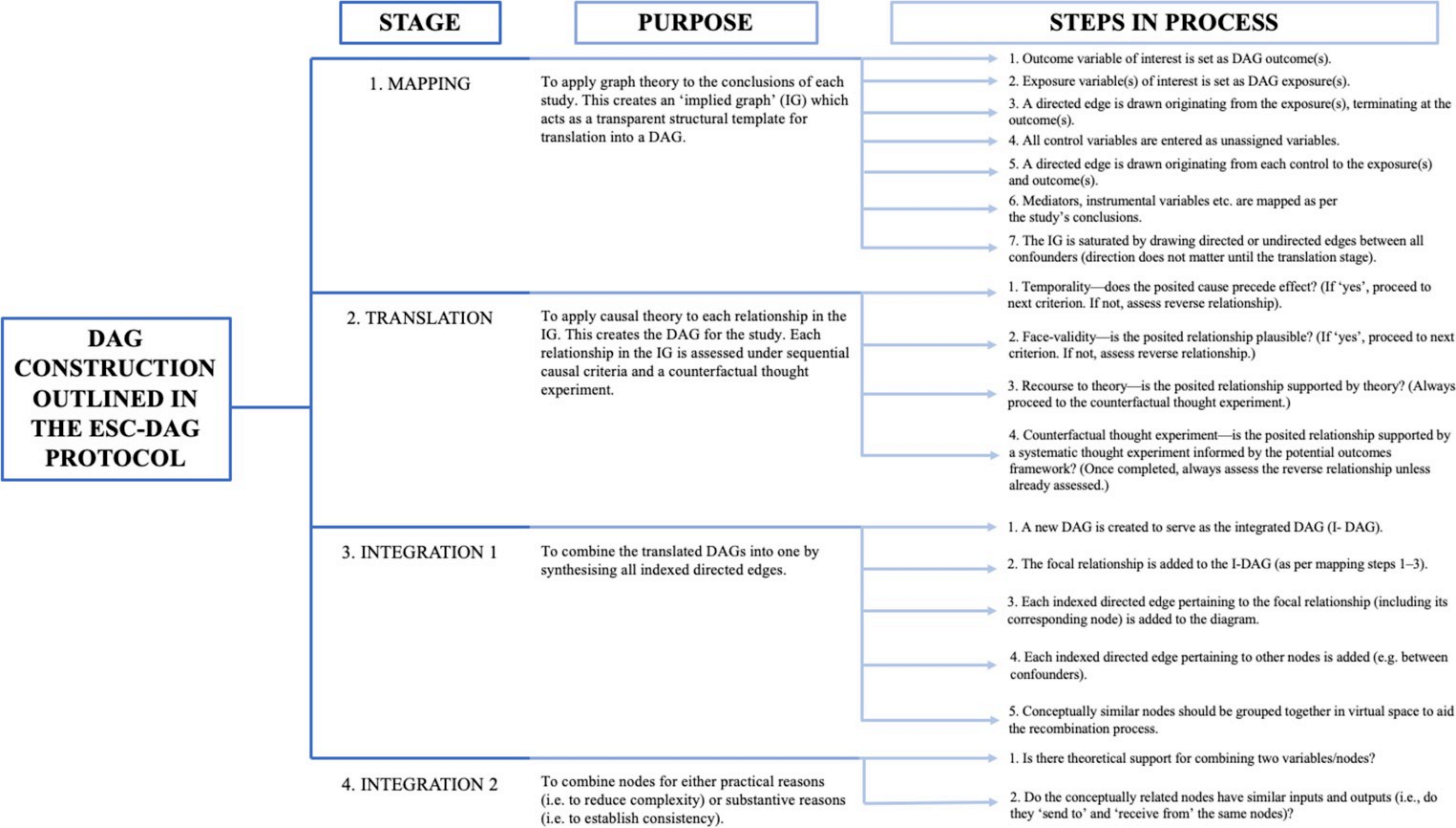
Stages, purpose, and the steps of DAG construction outlined in the ESC-DAG protocol [11] that the reviewers scored the final papers against. IG: implied graph (a DAG that acts as a structural template to build the DAG).

A modified inductive reasoning approach was taken to assess the nineteen studies included in the review [13]. This is outlined in Table 1. This approach was undertaken so we could follow a clear and logical framework to reach our final conclusions as a group. We used this method to qualitatively assess our studies in a transparent and repeatable way. The individual observation phase involved two authors scoring each study independently against the steps laid out in the ESC-DAG protocol. These stages and steps are depicted in Figs 2 and 3. This involved scoring the study either a 1 or 0 depending on whether the study had provided evidence that they had fulfilled that specific step. An initial agreement rate between the authors after individual observations was quantified using Cohen’s kappa statistic [14]. Group calibration was then undertaken to discuss the evidence for scoring each paper with a 1 or 0 against the specific steps of DAG construction. Unanimous group decisions about disagreements were made at this phase. A final group conclusion about the construction of DAGs within the surgical and perioperative literature was then made.

**Table 1:** Process followed by the authors to collect the data and score the DAGs in the studies against the ESC-DAG protocol [11].

		PROCESS FOLLOWED BY THE AUTHORS
**STAGE OF MODIFIED INDUCTIVE REASONING**	**INDIVIDUAL OBSERVATION**	Each DAG in the literature was scored against the ESC-DAG protocol with a 1 or a 0 depending on whether it had met the steps laid out in the ESC-DAG protocol.
	**GROUP CALIBRATION**	We then discussed findings, and a consensus was reached between all authors about the patterns of DAG construction between the studies included. Any disagreements were also debated.
	**FINAL DECISION**	We made a conclusion on the general construction of DAGs in this group of papers when scored against the ESC-DAG protocol.

The specific stages and steps we scored DAG construction against, as well as the purpose of each stage, are outlined in Figs 2 and 3. They are also discussed in detail in the ESC-DAG protocol [11].

A data collection form was constructed and reviewed by all authors before data extraction. The data collection form and individual observations of all nineteen papers can be found in S2 Appendix.

We also extracted key study characteristics including publication date relative to the ESC-DAG protocol and whether the study had included and discussed their handling of latent nodes. If the study incorporated time as a variable into their DAG, this was also recorded. This was so we could comment on how these aspects of DAG construction were undertaken, with reference to the ESC-DAG protocol.

Finally, each study was assessed by two authors independently against the Newcastle-Ottawa scale to assess potential risks of bias and to assess the quality of the final studies included [15]. Scores can be found in Table 2 and the full scoring can be found in S3 Appendix.

**Table 2:** Table depicting the year, journal, study objective and publication date relative to the ESC-DAG protocol of the final nineteen studies. Whether the DAG within the study included a temporal variable is also recorded. A study including temporal variables means this study acknowledged or specified that a certain variable e.g., drug usage was measured at a specific time point in the preoperative pathway.

Study	Year Published	Journal	Study Objective	Published after the ESC-DAG protocol?	Did the study include nodes with a temporal element?	Newcastle Ottawa Scale scoring for each study
Ferrando et al. [16]	2022	Acta Anaesthesiologica Scandinavica	To investigate whether an intraoperative open lung condition reduces the risk of developing a composite of postoperative pulmonary complications.	Y	Y	7
Kalkan et al. [17]	2022	Angiology	To determine whether the uric acid/albumin ratio is a predictor of mortality in STEMI patients.	Y	N	7
Lam et al. [18]	2022	Anaesthesia	To clarify the association between glycated haemoglobin and postoperative outcomes in people without an existing diagnosis of diabetes.	Y	N	7
Nimmaanrat et al. [19]	2022	BMC Anaesthesiology	To find the factors for predicting the amount of opioid requirement post-surgery.	Y	Y	7
Qian et al. [20]	2022	International journal of clinical practice	To clarify the efficiency and outcomes of suctioning ureteral access sheath during flexible ureteroscopic lithotripsy for the management of renal stones.	Y	Y	8
Steele et al. [21]	2022	Frontiers in human neuroscience	To quantify the causal effects of altered motor control and other impairments on gait, before and after single-event multi-level orthopaedic surgery.	Y	Y	7
Bedir et al. [22]	2021	Journal of Cancer Research and Clinical Oncology	To quantify the effect of socioeconomic inequality on head and neck cancer survival.	Y	N	8
Cagigas et al. [23]	2021	International Journal of Colorectal Disease	To predict intra-abdominal infections after colorectal surgery.	Y	Y	5
Laitinen et al. [24]	2021	Acta Orthopaedica	To investigate implant survival and complications of different surgical strategies in the treatment of proximal tibia pathological fractures.	Y	Y	6
Tetta et al. [25]	2021	Surgical Oncology	To predict lung recurrence and disease-specific mortality after pulmonary metastasectomy for soft tissue sarcoma.	Y	Y	6
Wang et al. [26]	2021	BMJ Open	To analyse the clinical value of primary site surgery in improving the cancer-specific survival and overall survival of initial metastatic cervical cancer patients.	Y	N	8
Duprey et al. [27]	2020	Journal of the American Geriatrics Society	To provide researchers with guidance on the methodological tools to use data from clinical cohorts to better understand medication risk factors and outcomes.	Y	N	8
Pollmann et al. [28]	2020	Acta Orthopaedica	To identify the contribution of early deep surgical site infection to mortality after hip fracture surgery.	Y	Y	8
Sittivarakul et al. [29]	2020	Medicine	To determine the surgical outcomes and prognostic factors of cytomegalovirus retinitis-related retinal detachment in acquired immune deficiency syndrome patients following vitrectomy.	Y	Y	8
Hoorntje et al. [30]	2019	Orthopaedic Journal of Sports Medicine	To investigate the extent and timing of return to work in the largest high tibial osteotomy cohort investigated for return to work and to identify prognostic factors for return to work after high tibial osteotomy.	Y	Y	8
Kerkhoffs et al. [31]	2019	The American Journal of Sports Medicine	To investigate the extent and timing of return to sport after high tibial osteotomy in the largest cohort investigated for return to sport to date and to identify prognostic factors for successful return to sport.	N	Y	7
Pathak et al. [32]	2018	Hospital paediatrics	To identify predictors of oophorectomy in girls hospitalized throughout Texas with ovarian torsion.	N	N	7
Asgari et al. [33]	2011	Archives of Dermatology	To identify preoperative, intraoperative, and postoperative variables that predict higher short and long-term patient satisfaction with Mohs surgery.	N	Y	7
Sehrndt et al. [34]	2011	Gesundheitswesen	To investigate the influence of socioeconomic status on health-related quality of life in patients before and after aortocoronary bypass surgery.	N	N	7

STEMI, ST-elevation myocardial infarction.

## Results

### Characteristics of the final studies included

We identified 638 citations after the removal of duplicates. After abstract and full text screening, nineteen (3%) were included in our final synthesis. We included one study published in German which was read and reviewed by two native speakers on our study team (KMD, KK). We did not include studies that sought to generate a Bayesian network.

Once abstracts had been screened; fifty-four studies remained and were assessed for eligibility by screening the full text and supplementary material for a DAG. After this round of full text screening, nineteen studies were included in the final review. Five of these nineteen studies [16, 17, 19–21] were identified during the updated search in June 2022. References of the final nineteen studies were also screened for relevant studies. One additional study was included via this method (Fig 1).

Although the terms Bayesian network and DAG can both be used in the literature to refer to a causal graphical model, Bayesian networks do not necessarily describe causal relationships and typically do not solely use expert opinion in their construction [35], they are therefore not explicitly covered by the ESC-DAG framework. However, one screened study [23] that constructed a Bayesian network was included, as the graphical core of their diagram describes causal relationships and was constructed solely from expert knowledge derived from literature and clinical practice. Therefore, we decided to include this study as the construction of this graph could have adhered to ESC-DAG protocol.

Of the final nineteen studies reviewed by two reviewers, the median number of stars obtained on the Newcastle-Ottawa scale was seven and the interquartile range was one. The lowest score on the Newcastle-Ottawa scale was five. The score of each paper is included in Table 2 and full scoring of all the nineteen papers can be found in S3 Appendix.

Table 2 shows the journals and years of publication for the studies included in this systematic review. Fifteen (79%) of the nineteen studies included were published after the ESC-DAG protocol [11] was available online on the 19^th^ of July 2019. No study referenced the ESC-DAG protocol.

Twelve studies (63%) included variables on their DAG with a temporal element. For instance, variables with relevant time dimensions such as ‘preoperative use of gabapentin’ [19].

We also assessed the geographical distribution of the final studies included. The studies were written by four authors affiliated in the USA (21%), two in China (11%), two in Germany (11%), two in Spain (11%), two in the Netherlands (11%), two in Thailand (11%), one in Turkey (5%), one in the United Kingdom (5%), one in Norway (5%), one in Finland (5%) and one in Italy (5%).

### Assessing DAG construction against the ESC-DAG protocol

Following individual observation and review of each paper, the initial agreement rate between reviewers was 0.86 (as calculated using a Cohen’s Kappa statistic). This suggests a high level of initial agreement between all reviewers when independently scoring the papers during the individual observation phase of our analysis [36].

Table 3 contains the individual scoring of each paper against the ESC-DAG protocol after a consensus was reached. It also demonstrates the patterns identified and general conclusion about the whole cohort of studies assessed. Even though no studies referenced the ESC-DAG protocol, studies did show evidence of fulfilling some stages and steps of the protocol. No studies showed evidence of completing any steps of mapping. Fifteen (79%) of the studies completed 50% or more of the translation and integration one steps. There was a large variation between the studies as to the number of steps fulfilled within each of these two stages. Two (11%) of the final studies fully completed integration stage two; no other studies completed any steps within this stag.

**Table 3:** Table demonstrating the results from the screening of all nineteen papers when assessed against the ESC-DAG protocol [11].

				PAPERS (Lead Author)																		
				Asgari [33]	Bedir [22]	Cagigas [23]	Duprey [27]	Ferrando [16]	Hoorntje [30]	Kalkan [17]	Kerkhoffs [31]	Laitinen [24]	Lam [18]	Nimmaanrat [19]	Pathak [32]	Pollmann [28]	Qian [20]	Sehrndt [34]	Sittivarakul [29]	Steele [21]	Tetta [25]	Wang [26]
**STAGE OF MODIFIED INDUCTIVE REASONING**	**INDIVIDUAL OBSERVATIONS**	**Percentage of steps in the ESC-DAG protocol each study has fulfilled during DAG construction**	Mapping (%)	0	0	0	0	0	0	0	0	0	0	0	0	0	0	0	0	0	0	0
			Translation (%)	75	100	75	75	75	75	25	62.5	0	50	50	50	25	75	50	75	62.5	0	50
			Integration 1 (%)	60	100	0	40	90	80	80	80	100	70	60	0	90	100	70	60	100	20	80
			Integration 2 (%)	0	100	0	0	0	0	0	0	0	0	0	0	0	100	0	0	0	0	0
	**GROUP CALLIBRATION**	**Key group observations on the construction of the DAG in all studies using the ESC-DAG protocol as guidance**		• No studies demonstrated any evidence of completing any steps of mapping.																		
				• Fifteen studies (79%) carried out 50% or more of the steps within the translation stage. The steps were discussed by some papers, but with strong variation amongst the information presented. Only one study showed evidence of performing a counterfactual thought experiment (one of the steps within this stage).																		
				• Fifteen studies (79%) undertook 50% or more of the steps of integration one; but did not discuss this in their text.																		
				• Only two studies (11%) undertook and discussed integration two.																		
	**FINAL DECISION**	**Overall conclusion of DAG construction from scoring against the ESC-DAG protocol**		No study displayed evidence of completely following the ESC-DAG protocol. Mapping (structured synthesis of previously published studies) was never documented. Overall, DAGs constructed in this selection of papers did not consistently adhere to the ESC-DAG protocol.																		

### Unmeasured variables

Table 4 demonstrates which studies acknowledged and discussed their methods for including unmeasured variables. Six studies (32%) did not include, acknowledge, or discuss any unmeasured variables. Seven studies (37%) acknowledged unmeasured variables within their study. However, none of these studies included these unmeasured variables in their DAG or discussed how unmeasured variables were managed or might impact on the interpretation of their findings. Two studies (11%) included unmeasured variables within their DAG. No study in these categories commented on how they tried to mitigate for any unmeasured variables, despite the acknowledgment that potentially key variables were not measured or incorporated in their DAG.

**Table 4:** Table demonstrating the extent to which different studies dealt with unmeasured variables, if at all.

Table demonstrating which studies included, acknowledged, and made corrections for unmeasured variables				
Unmeasured variables not mentioned	Unmeasured variables acknowledged; not included in the DAG or discussed in the study	Unmeasured variables included in the DAG; not discussed in the study	Unmeasured variables included in the DAG and discussed in the study	DAG constructed before data was collected; no unmeasured variables included
32% (n = 6)	37% (n = 7)	11% (n = 2)	16% (n = 3)	5% (n = 1)
Cagigas et al. [23]	Asgari et al. [33]	Hoorntje et al. [30]	Bedir et al. [22]	Sehrndt et al. [34]
Duprey et al. [27]	Ferrando et al. [16]	Kerkhoffs et al. [31]	Pollmann et al. [28]	
Kalkan et al. [17]	Laitinen et al. [24]		Pathak et al. [32]	
Lam et al. [18]	Qian et al. [20]			
Nimmaanrat et al. [19]	Sittivarakul et al. [29]			
Tetta et al. [25]	Steele et al. [21]			
	Wang et al. [26]			

Three studies (16%) included and discussed how unmeasured variables would affect their study. For instance, one study [22] aimed to quantify the effect of socioeconomic inequality on head and neck cancer survival. The authors acknowledged that human papillomavirus (HPV) status was an important unmeasured variable. Their approach was to infer HPV status based on tumour site (HPV-related sites vs HPV non-related sites), using another, measured variable to attempt to represent the uncaptured latent. This study referenced the scientific literature they used as evidence to make this inference.

One study [34] (5%) built their DAG and then collected conclusive data for their variables. This study therefore did not have any unmeasured variables.

## Discussion

In this systematic review, we provide the first comprehensive critique of the methods used in developing DAGs in perioperative and surgical research. Our main finding is that, at present, DAG construction does not consistently follow the newly published ESC-DAG protocol [11] and other elements of best practice [10]. This demonstrates the importance of newly emerging guidelines to strengthen practice in this area. Our findings are congruous with other literature regarding DAG usage and construction in observational studies [37] which could potentially limit the benefits of employing this approach if readers and reviewers cannot understand the assumptions that were used to underpin subsequent causal inferences.

Although our search strategy was not designed to generate a comprehensive denominator on the total number of conducted perioperative studies it is important to highlight the small number of studies included in our final synthesis (3% of initial citations), despite its comprehensiveness (three databases) and intended sensitivity. We found this surprising given the number of studies published in recent years that emphasise how important and useful ‘big data’ can and might be within this field [38–40]. No study specifically mentioned the ESC-DAG protocol, including fifteen studies that were published after the ESC-DAG protocol [11] was made available. This strongly suggests that no studies in our cohort were trying to explicitly follow this guidance and could reflect either a lack of awareness, a lag in its adoption into practice, or a lack of comfort in implementing and following its steps. However, all studies demonstrated evidence of fulfilling at least one or more of the stages and steps identified in the protocol.

We can only speculate as to why so few observational studies within this field utilise DAGs, especially as relevant methodological reviews have been published in the field of anaesthesia and surgery [8, 9, 41]. This may represent broader trends in biomedical observational research [10]. Alternatively, researchers may be aware of DAGs; but uncomfortable using these tools when they have a limited understanding of when and how to use them. However, more education and awareness may be required before researchers can use DAGs comfortably and to their full potential, especially because, as we have documented, there are currently few examples of such best-practice frameworks being implemented or reported in the literature.

Significantly, no study appeared to undertake the ‘mapping’ stage whereby a directed graph is drawn for relevant, previously published studies examining the same exposure and outcome relationships. Such a step would be a transparent way to communicate the controlling of ‘all known confounders’ that is often mentioned in observational research. It is plausible that the authors of the final nineteen studies built several template DAGs before arriving at the one presented in the study. However, none of these template DAGs were made available and thus the author’s thought processes cannot be followed. Therefore, the studies did not score anything when we were scoring them against the mapping stage of the protocol.

Fifteen of the studies (79%) carried out 50% or more of the translation stage. Translation exists to confirm which relationships in a template DAG depict a causal relationship. Our included studies demonstrated a strong variation in their discussion behind the relationships depicted in their DAG. Some studies did not qualify the causal relationships within their DAG at all [24, 25], whereas one study fulfilled all stages of translation and qualified the DAG they constructed robustly [22]. Given that a DAG is only as valid as the assumptions which underpin its construction, we would argue that even if authors are not following the ESC-DAG protocol, it is crucial that there is some discussion within a study that justifies the causal relationships depicted on a DAG. This allows readers to understand why specific relationships have been included and further understand and critique the statistical analysis within a study that the DAG guides. One strength of the DAG approach is that its robust mathematical underpinning is communicated to readers in an accessible pictorial format. However, if the rationale for its construction is not clearly communicated it is difficult for researchers to interpret results and critique findings based on the depicted relationships.

Seventeen studies (89%) undertook at least some steps of integration stage one but did not discuss this in their text. Integration one exists to combine the causal relationships identified in the translation stage into a DAG. During this stage authors build the final DAG that they will use within their study. Authors may not have discussed this stage within their text as they may have felt that they did not need to describe how they built their DAG from exposure to outcome, to the final DAG identifying all other causal relationships within their system.

Furthermore, only two studies (11%) undertook and discussed integration stage two. Integration stage two exists to combine nodes in the final DAG for either practical or substantive reasons. Like mapping, we believe studies could have undertaken integration two but may not have provided any evidence of completing them within the text. It is plausible that authors of the studies we analysed were combining nodes within their DAG. This is a step of the integration two stage in the protocol that only two studies demonstrated any evidence of following. However, we acknowledge that more studies may have combined nodes without making this explicit in the text. An example of this could include combining many health conditions within a study as a ‘co-morbidities’ node on a DAG. Direct communication of these assumptions would arguably be of use to reviewers or other researchers attempting to replicate findings in other settings.

The ESC-DAG protocol does not perhaps encompass all elements of best-practice. For instance, it does not make explicit mention to the inclusion or handling of unmeasured variables, a crucial step in ensuring a DAG is causally valid [4]. Explicitly depicting unmeasured variables on a DAG helps to highlight any potential sources of unobserved confounding. It also allows authors to fully communicate their causal model, which can further lead to debate and inform future research. Some studies included their unmeasured variables on their DAG but there was wide variation between the studies analysed. We found that some studies did not acknowledge any unmeasured variables whilst others incorporating unmeasured variables into their DAG and gave detailed discussion as to how they had attempted to control for these factors. One study [34] communicated that they had built their DAG before collecting conclusive data for all the variables included. It is ideal if a DAG can be constructed using variables that have data available for them. However, omitting variables from a DAG because authors believe they will not be able to collect conclusive data for a variable goes against best practice [10]. Further to the above, it is vital that those constructing DAGs include all variables, unmeasured or measured, that may have causal relationships to the system being studied.

Another additional element of best practice we examined was to understand how time was displayed or handled within published DAG studies. Perioperatively, the explicit mention of the time at which a variable is measured is vital, with a low pre-operative haemoglobin having a completely different interpretation and causal relationship than one immediately after surgery. A further relevant example of this may include the effect of arterial blood pressure (ABP) on three-day postoperative mortality. Intraoperative ABP pressure may have a different causal effect on mortality than preoperative ABP. Therefore, it is important researchers acknowledge how time may change their causal relationships depicted. We found that twelve studies (63%) acknowledged the time point of a variable within the group of nineteen studies analysed. Further to this, we thought that *Asgari et al*. [33] presented a very effective way to present time within a perioperative DAG, with nodes grouped according to ‘pre’, ‘intra’, and ‘post’ operative phases of care.

The ESC-DAG protocol gives researchers a strong framework to build DAGs. This is a clear development in the field where best practice for DAG construction did not exist [37]. However, its relatively recent publication means that a critique of its impact on transparency and reproducibility cannot yet be commented upon. Beyond the core stages of the protocol that we have discussed so far, the concept of a ‘directed edge index’ and ‘decision log’ to communicate assumptions around edge incorporation and direction could be a valuable tool to support the open-science framework. The appendix of the ESC-DAG protocol asks researchers to explicitly mention what their outcome, exposures, controls, and mediators are, to document their assumptions around edge inclusion, and fully communicate their rationale. Based on our literature findings we feel the use of such a reporting template (perhaps as supplemental material) would be useful in increasing the transparency of assumptions underlying causal analyses, in a manner analogous to the ‘target-trial’ [42] framework that is advocated to design a causally focused observational study.

However, to our knowledge we are not aware of any surgical and perioperative journal that requires observational studies to construct a DAG if they are seeking to make a causal interpretation. The New England Journal of Medicine (NEJM) [43] explicitly state that *‘causal language should not be used in observational studies where only associations can be estimated’*. However, The NEJM does not mention the DAG when discussing methods observational studies should use to explicitly discuss and estimate a causal effect. The guidance for authors publishing in the British Journal of Anaesthesia [44] and British Journal of Surgery [45] do not expect authors to have constructed DAGs. Both journals ask authors publishing observational studies to adhere to the Strengthening the Reporting of Observational studies in Epidemiology (STROBE) [46] guidelines. Further to this, the STROBE guidelines ask authors to ‘describe any efforts to address potential source of bias’ in the twenty-two-item checklist provided. However, there is no mention as to which methodological approach authors should use to deal with bias and there is no mention of DAGs. This further contributes to our point that the surgical and perioperative field may not be aware of how the use of a DAG could strengthen confidence in causal inferences within observational research.

Given the benefits of DAGs in guiding analysis, their greater use in the perioperative literature could arguably improve the robustness of studies seeking to draw causal inferences. Beyond this improved accuracy, submitting information justifying the reasons behind a constructed causal model could also improve communication of assumptions from researchers to readers. Future guidelines for observational studies from journals, requiring the reporting of a DAG and its constructions might be one way to increase uptake of the technique.

We feel our review has several strengths. Firstly, we used a broad search strategy to encompass all aspects of the surgical and perioperative literature, prospectively registered and followed our protocol, and sought to incorporate relevant aspects of best-practice beyond the newly published ESC-DAG framework. We had a strong agreement rate within our group prior to the making of a final consensus decision. This is denoted by a Cohen’s kappa statistic of 0.86 [36]. We do acknowledge potential limitations. Given DAGs are normally included in the methods or supplementary section of studies, some studies that did not mention the use of a DAG within their title/abstract/keywords may have been missed. We believe the method of including any abstract that had language pertaining to causal inference or DAGs minimised the risk of missing eligible studies that had constructed a DAG, as we expected authors to draw attention to the use of this tool in the title or abstract. However, we acknowledge that a small number of studies may have still been missed if key terms were not included in the abstract or title. This should not detract from the validity of the critique of those studies that we did identify. We also acknowledge that there was a small sample size of final studies to screen against the ESC-DAG protocol and without a clear denominator as to the number of observational studies conducted in the perioperative literature in this period it is difficult to truly judge the prevalence of DAGs within the field. However, our primary focus during this study was to assess DAGs constructed in the literature against the ESC-DAG protocol, rather than assess their prevalence. A further study could address this question in greater depth. The ESC-DAG framework specifically deals with synthesising published literature into a causal model. However, expert opinion is a crucial component of such a process and studies have started to look at how qualitative techniques can be used to distil this expertise into a DAG [47]. However, numerous studies cited the literature in their DAG and so even if a mixed methods approach was employed, the use of the ‘mapping’ phase would still be useful in communicating the findings of previously conducted studies. Finally, we recognise that other frameworks for DAG construction are also being published and thus the field is an emerging one. The DAG With Omitted Objects Displayed (DAGWOOD) [48] framework is another framework that can be used for generating causal models and one that reinforces best DAG practices. We did not assess our final studies against this recently published framework. However, given the quality of reporting DAG construction in our studies; we suspect that papers would still not do well when compared against this or other DAG construction frameworks. More work is required to understand what an appropriate reporting framework is for both authors and readers.

## Conclusion

DAGs in the surgical and perioperative observational literature do not currently follow the ESC-DAG protocol or other areas of best practice. This has implications on transparency and reproducibility within the field but may be driven by the recency of publication or a lack of awareness. Further improvements to DAG construction and reporting within the perioperative and surgical literature will aid in reader interpretation.

## Supporting information

S1 Checklist Completed PRISMA checklist for this systematic review. (DOCX)

S1 Appendix Search strategies used to identify relevant articles. (DOCX)

S2 Appendix Individual scores for each study when scored against the ESC-DAG protocol [11]. (XLSX)

S3 Appendix Individual scores for each study when scored against the Newcastle-Ottawa Scale [15]. (XLSX)
