# Correction: Directed acyclic graphs in perioperative observational research–A systematic review and critique against best practice recommendations

**DOI:** 10.1371/journal.pone.0334522

**Published:** 2025-10-13

**Authors:** Matthew Lamont Watson, Sebastian H. M. Hickman, Kaya Marlen Dreesbeimdiek, Katharina Kohler, Daniel J. Stubbs

In Table 2, there are errors in the Newcastle Ottawa scores used to quantify the quality of included studies. The score for the studies Kalkan et al. [17], Lam et al. [18], Nimmaanrat et al. [19], Bedir et al. [22], Cagigas et al. [23], Laitinen et al. [24], Tetta et al. [25], Wang et al. [26], Pollmann et al. [28], Sittivarakul et al. [29], Hoorntje et al. [30], Kerkhoffs et al. [31], Pathak et al. [32], Asgari et al. [33] reflected in the column Newcastle Ottawa Scale scoring for each study should increase one star. Also, the statement ‘Newcastle Ottawa scores are also shown for each study. STEMI, ST-elevation myocardial infarction.’ in its caption should have been included. Please see the correct Table 2 and its caption here.

**Table 2:** Table depicting the year, journal, study objective and publication date relative to the ESC-DAG protocol of the final nineteen studies. Whether the DAG within the study included a temporal variable is also recorded. A study including temporal variables means this study acknowledged or specified that a certain variable, e.g., drug usage was measured at a specific time point in the preoperative pathway. Newcastle Ottawa scores are also shown for each study. STEMI, ST-elevation myocardial infarction.

Study	Year Published	Journal	Study Objective	Published after the ESC-DAG protocol?	Did the study include nodes with a temporal element?	Newcastle Ottawa Scale scoring for each study
Ferrando et al. [16]	2022	Acta Anaesthesiologica Scandinavica	To investigate whether an intraoperative open lung condition reduces the risk of developing a composite of postoperative pulmonary complications.	Y	Y	7
Kalkan et al. [17]	2022	Angiology	To determine whether the uric acid/albumin ratio is a predictor of mortality in STEMI patients.	Y	N	8
Lam et al. [18]	2022	Anaesthesia	To clarify the association between glycated haemoglobin and postoperative outcomes in people without an existing diagnosis of diabetes.	Y	N	8
Nimmaanrat et al. [19]	2022	BMC Anaesthesiology	To find the factors for predicting the amount of opioid requirement post-surgery.	Y	Y	8
Qian et al. [20]	2022	International journal of clinical practice	To clarify the efficiency and outcomes of suctioning ureteral access sheath during flexible ureteroscopic lithotripsy for the management of renal stones.	Y	Y	8
Steele et al. [21]	2022	Frontiers in human neuroscience	To quantify the causal effects of altered motor control and other impairments on gait, before and after single-event multi-level orthopaedic surgery.	Y	Y	7
Bedir et al. [22]	2021	Journal of Cancer Research and Clinical Oncology	To quantify the effect of socioeconomic inequality on head and neck cancer survival.	Y	N	9
Cagigas et al. [23]	2021	International Journal of Colorectal Disease	To predict intra-abdominal infections after colorectal surgery.	Y	Y	6
Laitinen et al. [24]	2021	Acta Orthopaedica	To investigate implant survival and complications of different surgical strategies in the treatment of proximal tibia pathological fractures.	Y	Y	7
Tetta et al. [25]	2021	Surgical Oncology	To predict lung recurrence and disease-specific mortality after pulmonary metastasectomy for soft tissue sarcoma.	Y	Y	7
Wang et al. [26]	2021	BMJ Open	To analyse the clinical value of primary site surgery in improving the cancer-specific survival and overall survival of initial metastatic cervical cancer patients.	Y	N	9
Duprey et al. [27]	2020	Journal of the American Geriatrics Society	To provide researchers with guidance on the methodological tools to use data from clinical cohorts to better understand medication risk factors and outcomes.	Y	N	8
Pollmann et al. [28]	2020	Acta Orthopaedica	To identify the contribution of early deep surgical site infection to mortality after hip fracture surgery.	Y	Y	9
Sittivarakul et al. [29]	2020	Medicine	To determine the surgical outcomes and prognostic factors of cytomegalovirus retinitis-related retinal detachment in acquired immune deficiency syndrome patients following vitrectomy.	Y	Y	9
Hoorntje et al. [30]	2019	Orthopaedic Journal of Sports Medicine	To investigate the extent and timing of return to work in the largest high tibial osteotomy cohort investigated for return to work and to identify prognostic factors for return to work after high tibial osteotomy.	Y	Y	9
Kerkhoffs et al. [31]	2019	The American Journal of Sports Medicine	To investigate the extent and timing of return to sport after high tibial osteotomy in the largest cohort investigated for return to sport to date and to identify prognostic factors for successful return to sport.	N	Y	8
Pathak et al. [32]	2018	Hospital paediatrics	To identify predictors of oophorectomy in girls hospitalized throughout Texas with ovarian torsion.	N	N	8
Asgari et al. [33]	2011	Archives of Dermatology	To identify preoperative, intraoperative, and postoperative variables that predict higher short and long-term patient satisfaction with Mohs surgery.	N	Y	8
Sehrndt et al. [34]	2011	Gesundheitswesen	To investigate the influence of socioeconomic status on health-related quality of life in patients before and after aortocoronary bypass surgery.	N	N	7

Consequently, in S3 Appendix, there are errors in the values reflected in the Total Score and % given 9 points (stars) available column. Please see the correct S3 Appendix below.

Moreover, in the Characteristics of the final studies included subsection of Results, the fourth paragraph is incorrect. The correct paragraph is: Of the final nineteen studies reviewed by two reviewers, the median number of stars obtained on the Newcastle-Ottawa score was eight and the interquartile range was one and a half. The lowest score on the Newcastle-Ottawa scale was six. The score of each paper is included in Table 2 and full scoring of all the nineteen papers can be found in S3 Appendix.

## Supporting information

S3 Appendix (XLSX)
