# Supplementary material for: Directed acyclic graphs in perioperative observational research–A systematic review and critique against best practice recommendations
Source: PLoS One. 2023 Feb 9;18(2):e0281259. doi: 10.1371/journal.pone.0281259 (PMC9910726; doi:10.1371/journal.pone.0281259)

­­­­­Systematic Search: Directed Acyclic Graphs (DAGs) in the Surgical and Perioperative Literature (30/06/2022)

OVID to search Embase

<https://authproxy.bma.org.uk/process/redirects?url=https://ovidsp.ovid.com/ovidweb.cgi?T=JS&NEWS=N&PAGE=main&SHAREDSEARCHID=2Lwk9aE9wYi4qELkyqIj8cTdFMxl3oaEuZTrUJ7aVITwkTJxMRCXn6A4nezPPPAsD>

Embase <1974 to 2022 June 29>

1 exp directed acyclic graph/ 2882

2 DAG.mp. 5687

3 dags.mp. 832

4 directed acyclic graph.mp. 969

5 directed a-cyclic graph.mp. 1

6 directed acyclic network.mp. or directed acyclic graph/ 519

7 directed acyclical graph.mp. or directed acyclic graph/ 513

8 exp surgery/ 5393883

9 exp perioperative medicine/ 467

10 exp anesthesiology/ 20051

11 exp anesthesiology software/ 61

12 exp anesthesiology diagnostic device/ 33249

13 exp causal modeling/ 1382

14 1 or 2 or 3 or 4 or 5 or 6 or 7 or 13 10605

15 8 or 9 or 10 or 11 or 12 5432193

16 14 and 15 563

OVID to search Medline

<https://authproxy.bma.org.uk/process/redirects?url=https://ovidsp.ovid.com/ovidweb.cgi?T=JS&NEWS=N&PAGE=main&SHAREDSEARCHID=6q47RSU4OfKeiwmUNZDgfxJOknzjNqTJZDvsUwbfNKGkGUILDlhHRwh9MwzkWvdks>

Ovid MEDLINE(R) ALL <1946 to June 29, 2022>

1 exp Specialties, Surgical/ 214117

2 exp "Anesthesia and Analgesia"/ 246945

3 exp Surgical Procedures, Operative/ 3438011

4 2 or 3 3572696

5 exp Anesthesiology/ 32754

6 exp Anesthesia/ 201983

7 5 or 6 217892

8 exp Perioperative Medicine/ 104

9 directed acyclic graph.mp. 575

10 DAG.mp. 4861

11 exp Diacylglycerol Kinase/ 1289

12 exp Diglycerides/ 7631

13 exp Signal Transduction/ 659284

14 DAGS.mp. 762

15 directed a-cyclic graph.mp. 1

16 directed acyclic network.mp. 11

17 directed acyclical graph.mp. 2

18 causal diagram.mp. 77

19 structural causal model.mp. 21

20 exp Protein Kinase C/ 45908

21 11 or 12 or 13 or 20 696092

22 (10 or 14) not 21 2663

23 9 or 15 or 16 or 17 or 18 or 19 or 22 3130

24 1 or 4 or 7 or 8 3729736

25 23 and 24 123

COCHRANE


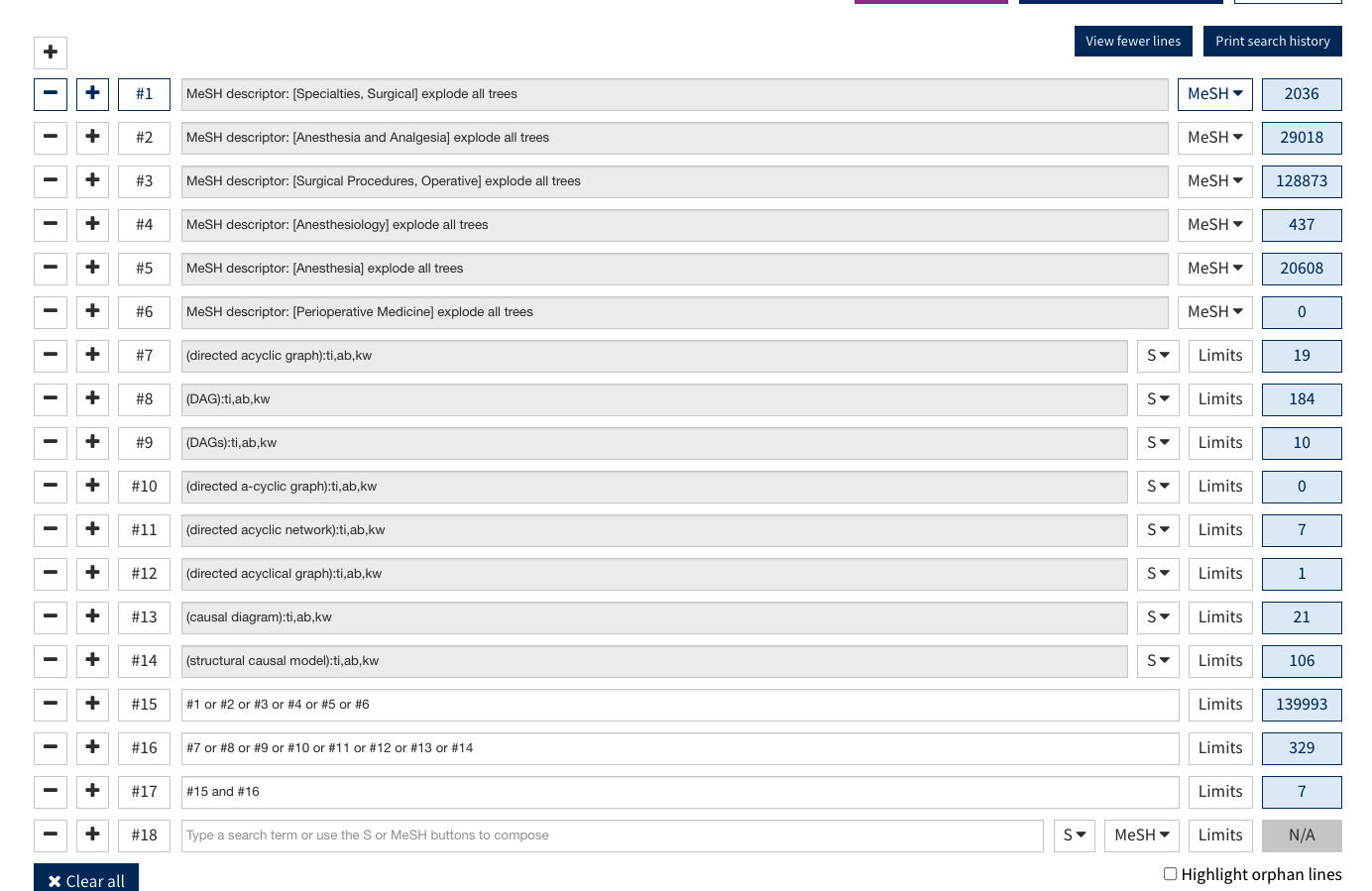

Supplement: S1 Appendix — (DOCX) [file pone.0281259.s002.docx]
